# Improvement in diagnostic delays over time in patients with hereditary angioedema: findings from the Icatibant Outcome Survey

**DOI:** 10.1186/s13601-018-0229-4

**Published:** 2018-10-12

**Authors:** Andrea Zanichelli, Markus Magerl, Hilary J. Longhurst, Werner Aberer, Teresa Caballero, Laurence Bouillet, Anette Bygum, Anete S. Grumach, Jaco Botha, Irmgard Andresen, Marcus Maurer, the IOS Study Group

**Affiliations:** 1 Department of Biomedical and Clinical Sciences Luigi Sacco, University of Milan, ASST Fatebenefratelli Sacco, Milan, Italy; 2 Department of Dermatology and Allergy, Allergie-Centrum-Charité, Charité–Universitätsmedizin Berlin, Berlin, Germany; 3 Department of Immunology, Barts Health NHS Trust, London, UK; 4 Department of Dermatology and Venereology, Medical University of Graz, Graz, Austria; 5 Department of Allergy, Hospital La Paz Institute for Health Research (IdiPaz), Biomedical Research Network on Rare Diseases (CIBERER, U754), Madrid, Spain; 6 National Reference Centre for Angioedema, Internal Medicine Department, Grenoble University Hospital, Grenoble, France; 7 Department of Dermatology and Allergy Centre, Odense University Hospital, Odense, Denmark; 8 Faculdade de Medicina ABC, São Paulo, Brazil; 9 Shire, Zug, Switzerland; 10 Present Address: Department of Clinical Biochemistry and Immunology, Addenbrooke’s Hospital, Cambridge University Hospitals NHS Foundation Trust, Cambridge, UK

**Keywords:** Hereditary angioedema, HAE, C1-INH-HAE, Diagnosis, Delay in diagnosis

## Abstract

The objective of this analysis was to evaluate the change over time in age at first symptoms, age at diagnosis, and delay in diagnosis using data from the Icatibant Outcome Survey (IOS). Patients with a diagnosis of C1-INH-HAE who were born before the year 1990 and who were diagnosed before they reached 25 years of age were included in the analysis. Both age at diagnosis and delay in diagnosis of C1-INH-HAE appear to decline with later decade of birth, despite wide variation across the countries assessed, suggesting that improved disease awareness causes increased rates of earlier diagnosis over time. Our findings demonstrate that some patients are still experiencing long delays to diagnosis, indicating an ongoing need for improved disease awareness.

To the Editor,

Hereditary angioedema due to C1 inhibitor deficiency or dysfunction (type I or type II; C1-INH-HAE) is a rare genetic disease characterized by repeated episodes of bradykinin-mediated swelling in subcutaneous or submucosal tissues [1, 2]. C1-INH-HAE is often poorly recognized because of its nonspecific signs and symptoms. As a result, misdiagnoses and delays in obtaining a correct diagnosis are common [3, 4]. The impact of a late diagnosis can be high, as initiation of appropriate therapy is delayed, putting patients at increased risk of morbidity and mortality [4, 5] and leading to unnecessary medical or surgical procedures [6]. Given the young age of patients at symptom onset, a delayed diagnosis may cause disruption of education or early working life, and significantly impacts quality of life and social involvement [7, 8]. Although awareness of C1-INH-HAE has improved over recent decades, it is not clear if this has translated into earlier diagnosis. Here, we evaluated the change in age at first symptoms, age at diagnosis, and delay in diagnosis by decade of birth in patients with C1-INH-HAE enrolled in the Icatibant Outcome Survey (IOS). IOS is an ongoing, international, multicenter, prospective, observational study (NCT01034969) designed to monitor the safety and effectiveness of icatibant, a bradykinin B_2_ receptor antagonist [9].

As of January 2017, 11 countries (Austria, Brazil, Czech Republic, Denmark, France, Germany, Greece, Israel, Italy, Spain, and the United Kingdom) were involved in the registry. Participation is voluntary, at the discretion of the physician and the patient, and patients are managed under the direction of their physician in accordance with routine clinical practice. Patient data, including year of birth, age at diagnosis, and delay between symptom onset and diagnosis, are collected by physicians using a web-based electronic case report form at the time of enrollment, and during subsequent routine examinations or visits to manage angioedema attacks. Written informed consent is obtained from all patients prior to enrollment in IOS. IOS is conducted in accordance with the Declaration of Helsinki and the International Conference on Harmonization Good Clinical Practice Guidelines, and with approval from local ethics committees and/or health authorities.

This analysis included patients with C1-INH-HAE who were born before 1990 and diagnosed before the age of 25 years. Patients diagnosed before the onset of symptoms, owing to a family history of C1-INH-HAE, were excluded. Data were collected from July 2009 through January 2017. Owing to the observational nature of the registry, all analyses were considered exploratory and no adjustment for multiplicity was performed. Linear regression analyses were performed to determine the correlation between time to event parameters and decade of birth, with a statistical significance level of α = 0.05.

As of January 2017, 250 patients with C1-INH-HAE type I (n = 240) or type II (n = 10) enrolled in IOS met the inclusion criteria. Of these, 139 (55.6%) were female. Median age at onset of symptoms was 9.0 years (interquartile range [IQR]: 4.0–16.0 years), median age at diagnosis was 16.7 years (IQR: 10.1–19.8 years), and median delay in diagnosis was 2.6 years (IQR: 0.1–9.7 years; Fig. 1a–c). There was considerable variation among countries, with median age at onset of symptoms ranging from 0.5 to 12.0 years, median age at diagnosis ranging from 13.5 to 22.3 years, and median delay in diagnosis ranging from 0.13 to 17.3 years (Fig. 1a–c).

**Fig. 1:**
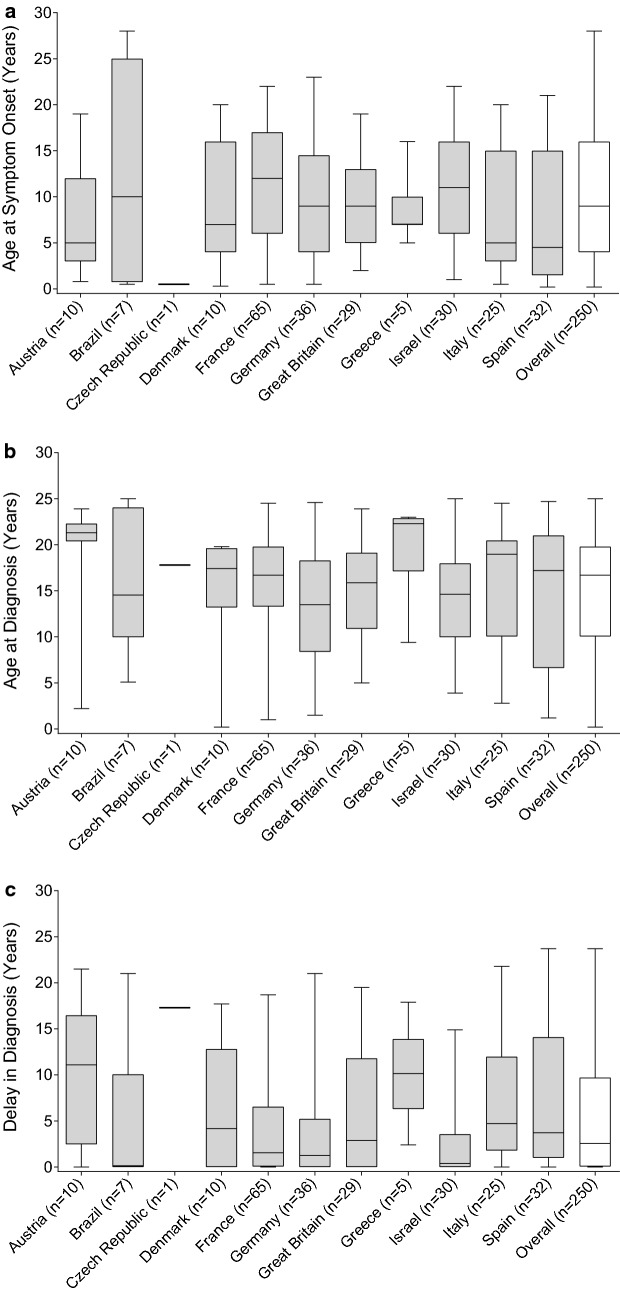
**a** Age at symptom onset by country. **b** Age at diagnosis by country. **c** Delay in diagnosis by country. The horizontal line inside each box indicates the median, the lower and upper borders of each box indicate the first and third quartiles, and the lower and upper box whiskers indicate the minimum and maximum values

Using linear regression analysis, we found that age at diagnosis and delay in diagnosis both declined with later decade of birth (*p *≤ 0.0001, Pearson correlation coefficient r = − 0.2659; and *p *= 0.0029, r = − 0.1874, respectively). Patients born during 1950–1960 (n = 24) were diagnosed at a median age of 20.2 years compared with 15.4 years for those born during 1980–1990 (n = 94), whereas patients born during 1950–1960 experienced a delay in diagnosis of 7.0 years compared with 1.4 years for those born during 1980–1990. Age at symptom onset remained unchanged irrespective of decade of birth (Fig. 2a–c). Patients with a family history of C1-INH-HAE (n = 180; 72.0%) had a median delay in diagnosis of 2.0 years compared with 5.6 years for those with no or unknown family history (*p *= 0.0092).

**Fig. 2:**
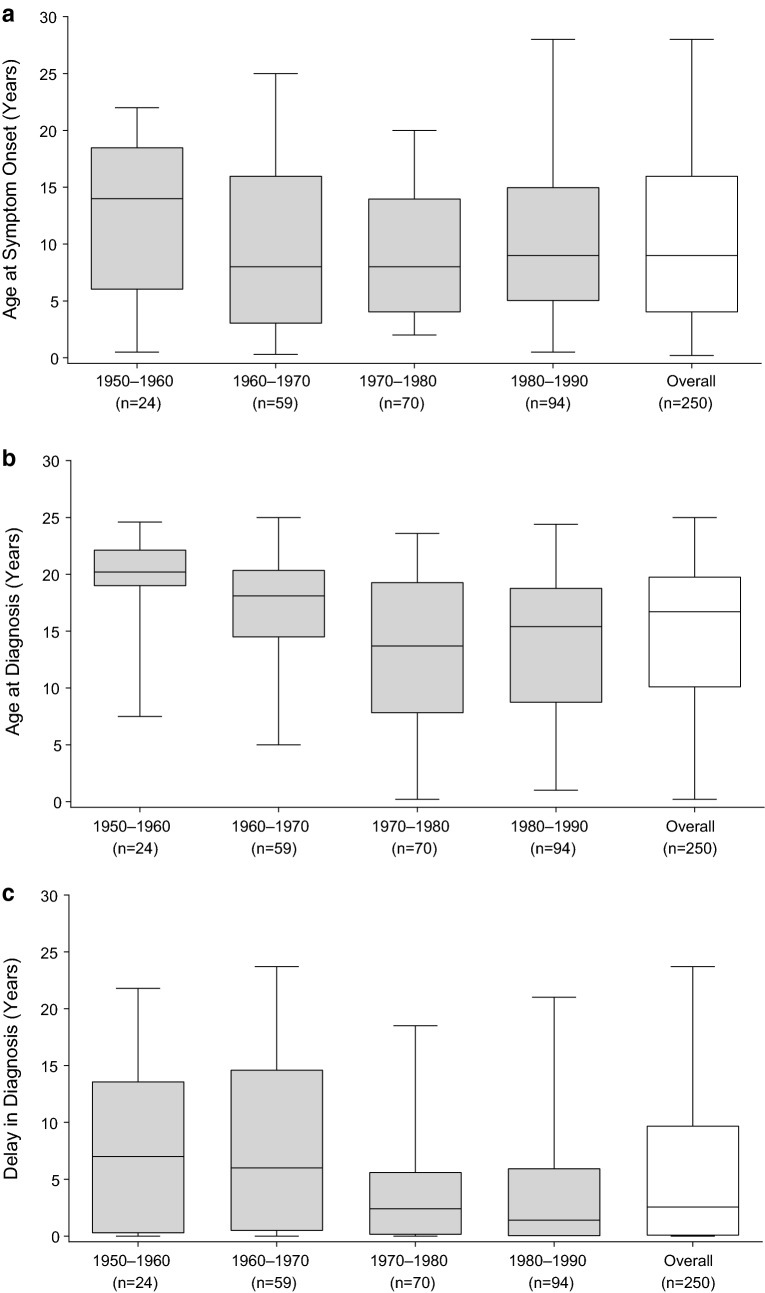
**a** Age at symptom onset by decade of birth. **b** Age at diagnosis by decade of birth. **c** Delay in diagnosis by decade of birth. The horizontal line inside each box indicates the median, the lower and upper borders of each box indicate the first and third quartiles, and the lower and upper box whiskers indicate the minimum and maximum values

Our findings demonstrate improvements in C1-INH-HAE diagnosis over time, with patients now more frequently being diagnosed at a younger age, and with shorter delays between symptom onset and diagnosis. However, patients diagnosed prior to symptom onset were excluded from this analysis, precluding the evaluation of diagnosis rates for those with a family history of HAE. Though data from registries such as IOS provide a valuable source of real-world information, the voluntary nature of participation presents a number of limitations, including missing or incomplete data and potential selection bias. Almost two-thirds of patients included in this analysis were born between 1970 and 1990, suggestive of a potential bias towards recently diagnosed patients. Of note, some patients are still experiencing long delays to diagnosis, indicating an ongoing need for improved disease awareness.

## Abbreviations

C1-INH-HAE: hereditary angioedema due to C1 inhibitor deficiency or dysfunction
HAE: hereditary angioedema
IOS: Icatibant Outcome Survey
IQR: interquartile range

## References

[1] Longhurst H, Cicardi M (2012). Hereditary angio-oedema. Lancet.

[2] Maurer M, Magerl M, Ansotegui I, Aygoren-Pursun E, Betschel S, Bork K (2018). The international WAO/EAACI guideline for the management of hereditary angioedema-The 2017 revision and update. Allergy..

[3] Christiansen SC, Davis DK, Castaldo AJ, Zuraw BL (2016). Pediatric hereditary angioedema: onset, diagnostic delay, and disease severity. Clin Pediatr (Phila).

[4] Zanichelli A, Longhurst HJ, Maurer M, Bouillet L, Aberer W, Fabien V (2016). Misdiagnosis trends in patients with hereditary angioedema from the real-world clinical setting. Ann Allergy Asthma Immunol.

[5] Bork K, Hardt J, Witzke G (2012). Fatal laryngeal attacks and mortality in hereditary angioedema due to C1-INH deficiency. J Allergy Clin Immunol.

[6] Henao MP, Kraschnewski JL, Kelbel T, Craig TJ (2016). Diagnosis and screening of patients with hereditary angioedema in primary care. Ther Clin Risk Manag.

[7] Caballero T, Prior N (2017). Burden of illness and quality-of-life measures in angioedema conditions. Immunol Allergy Clin North Am.

[8] Longhurst H, Bygum A (2016). The humanistic, societal, and pharmaco-economic burden of angioedema. Clin Rev Allergy Immunol.

[9] Maurer M, Aberer W, Bouillet L, Caballero T, Fabien V, Kanny G (2013). Hereditary angioedema attacks resolve faster and are shorter after early icatibant treatment. PLoS ONE.

